# Induction of Heat Shock Protein 70 Ameliorates Ultraviolet-Induced Photokeratitis in Mice

**DOI:** 10.3390/ijms14012175

**Published:** 2013-01-22

**Authors:** Anton Lennikov, Nobuyoshi Kitaichi, Satoru Kase, Kousuke Noda, Yukihiro Horie, Akira Nakai, Shigeaki Ohno, Susumu Ishida

**Affiliations:** 1 Laboratory of Ocular Cell Biology and Visual Science, Department of Ophthalmology, Hokkaido University Graduate School of Medicine, Sapporo 060-8638, Japan; E-Mails: lennikov@gmail.com (A.L.); kaseron@aol.com (S.K.); nodako@med.hokudai.ac.jp (K.N.); y-horie@crux.ocn.ne.jp (Y.H.); ishidasu@med.hokudai.ac.jp (S.I.); 2 Department of Ocular Inflammation and Immunology, Hokkaido University Graduate School of Medicine, Sapporo 060-8638, Japan; E-Mail: sohno@med.hokudai.ac.jp; 3 Department of Ophthalmology, Health Sciences University of Hokkaido, Sapporo 002-8072, Japan; 4 Department of Biochemistry and Molecular Biology, Yamaguchi University School of Medicine, Ube 755-8505, Japan; E-Mail: anakai@yamaguchi-u.ac.jp

**Keywords:** GGA, geranylgeranylacetone, HSP, HSP70, UVB, keratitis, cornea, apoptosis

## Abstract

Acute ultraviolet (UV) B exposure causes photokeratitis and induces apoptosis in corneal cells. Geranylgeranylacetone (GGA) is an acyclic polyisoprenoid that induces expression of heat shock protein (HSP)70, a soluble intracellular chaperone protein expressed in various tissues, protecting cells against stress conditions. We examined whether induction of HSP70 has therapeutic effects on UV-photokeratitis in mice. C57 BL/6 mice were divided into four groups, GGA-treated (500 mg/kg/mouse) and UVB-exposed (400 mJ/cm^2^), GGA-untreated UVB-exposed (400 mJ/cm^2^), GGA-treated (500 mg/kg/mouse) but not exposed and naive controls. Eyeballs were collected 24 h after irradiation, and corneas were stained with hematoxylin and eosin (H&E) and terminal deoxynucleotidyl transferase dUTP nick end labeling (TUNEL). HSP70, reactive oxygen species (ROS) production, nuclear factor kappa-light-chain-enhancer of activated B cells (NF-κB) and protein kinase B (Akt) expression were also evaluated. Irradiated corneal epithelium was significantly thicker in the eyes of mice treated with GGA compared with those given the vehicle alone (*p* < 0.01). Significantly fewer TUNEL-positive cells were observed in the eyes of GGA-treated mice than controls after irradiation (*p* < 0.01). Corneal HSP70 levels were significantly elevated in corneas of mice treated with GGA (*p* < 0.05). ROS signal was not affected by GGA. NF-κB activation was reduced but phospho-(Ser/Ther) Akt substrate expression was increased in corneas after irradiation when treated with GGA. GGA-treatment induced HSP70 expression and ameliorated UV-induced corneal damage through the reduced NF-κB activation and possibly increased Akt phosphorilation.

## 1. Introduction

Ultraviolet (UV) irradiation is one of the several environmental hazards that may cause inflammatory reactions in ocular tissues. In recent years the decrease in atmospheric ozone has increased the chances of photochemically-induced ocular damage that previously had been limited to professional accidents when protective gear glasses were improperly used or not used at all [1]. Prolonged eye exposure to UVB (320 nm–280 nm) induces photokeratitis that affects all tissues of the cornea [2] and causes expression of various inflammatory agents including nuclear factor kappa-light-chain-enhancer of activated B cells (NF-κB), Prostaglandin E2 (PGE2), and others [1]. UVB exposure affects all tissues of the cornea [2] and causes apoptosis in corneal cells by direct cell membrane damage, deoxyribonucleic acid (DNA) damage, reactive oxygen species (ROS) induction, as well as a result of an inflammatory reaction.

Geranylgeranylacetone (GGA) is an acyclic polyisoprenoid widely used in Japan as an antiulcer drug. GGA reduces gastric mucosa damage and ulcer formation without affecting secretion of gastric acid or pepsin [3]. GGA induces heat shock protein (HSP)70 expression, as reported previously [4]. HSPs are soluble intracellular proteins expressed in various tissues, including the eyes [5]. We previously demonstrated that GGA administration had protective effects against ocular inflammatory disorders such as ischemia-induced retinal injury and experimental autoimmune uveoretinitis (EAU) in murine models [6,7]. Under stress conditions, HSPs are increased and function as molecular chaperones that protect proteins from damage or refold defective polypeptides in an attempt to restore their native conformation [8]. The HSPs are reported to protect cells against inflammation, infection, and autoimmune reactions [9,10]. A retinal phototoxicity study in rats showed that hyperthermic preconditioning contributed to retinal protection against light-induced photoreceptor degeneration [11]. In experiments with gene-modified drosophila, tissues from flies with up-regulated expression of HSP had fewer apoptotic changes after UV irradiation compared to naive flies [12].

Recent reports indicated that HSP70 activated protein kinase B (Akt) phosphorylation, inhibiting dephosphorylation and further activation of cell death pathways in the photorecepter cells of the eye [13] and ameliorated H_2_O_2_-induced apoptosis of corneal epithelial cells by suppressing caspase-3 and caspase-9 *in vitro* [14]. Therefore, HSP70 not only saves important components of the cell proteins but also directly saves the cell as whole. However, the relationship between HSP70 expression and corneal damage is not known.

In the present study, we investigated whether oral administrations of GGA induced a protective effect against UV-induced corneal damage in mice.

## 2. Results and Discussion

### 2.1. Hematoxylin and Eosin (H&E) and Terminal Deoxynucleotidyl Transferase dUTP Nick End Labeling (TUNEL) Staining

**Figure 1:**
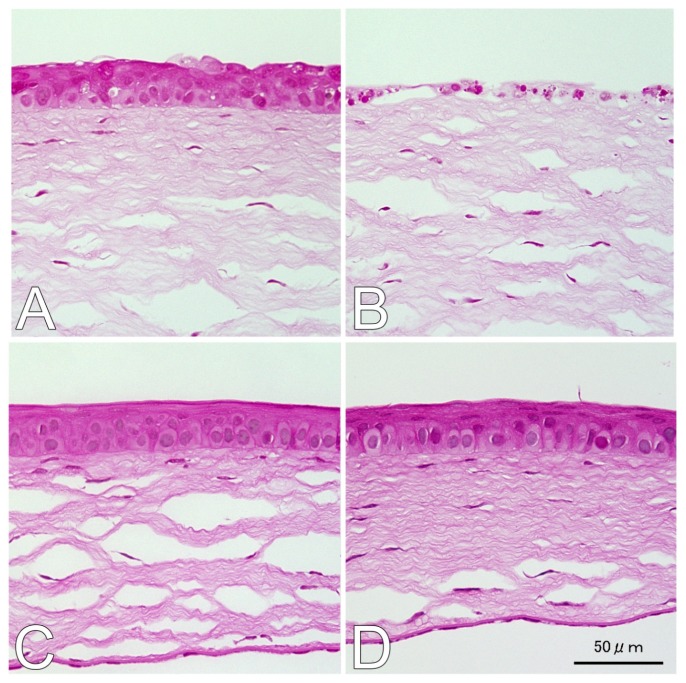

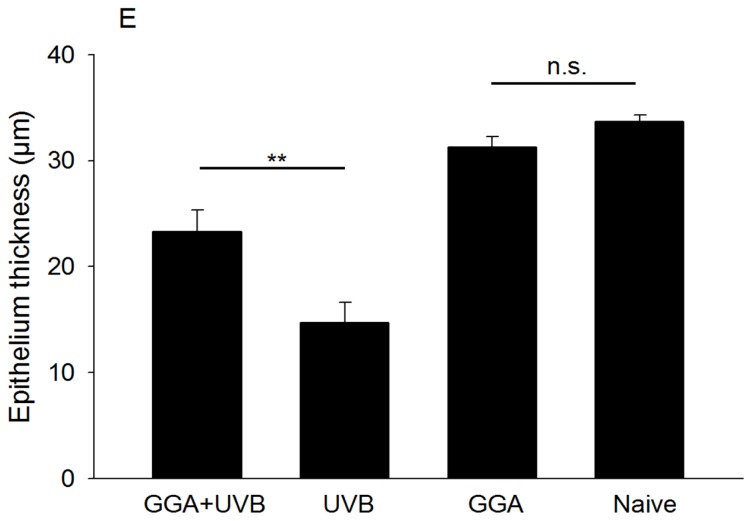
Morphological properties of ultraviolet (UV) B-irradiated corneas (**A**) Irradiated eyes of mice treated with geranylgeranylacetone (GGA); (**B**) Irradiated eyes of mice without GGA administration; (**C**) Unirradiated eyes of mice administered with GGA; (**D**) Naive corneas; (**E**) Mean corneal epithelium thickness. Data are shown as mean ± SEM (*n* = 10), **p < 0.01; n.s. *p* > 0.05 (Mann-Whitney U-Test).

Twenty-four hours after UVB irradiation, at a dose of 400 mJ/cm^2^, the corneal epithelium was well preserved and the thickness remained close to naive controls in mice treated with 500 mg/kg GGA (Figure 1A). However, thinning of the corneal epithelial layer was observed in the eyes of mice not treated with GGA after UV exposure (Figure 1B). The corneas of mice administered GGA without UVB irradiation (Figure 1C) showed no differences from naive corneas (Figure 1D). The mean values of the corneal epithelium thicknesses were calculated. The mean thicknesses were 22.3 ± 2.0 μm in the eyes of mice of the (GGA + UVB) group treated with GGA and UVB irradiation; 14.7 ± 2.0 μm after UVB-exposure in the eyes of the mice not treated with GGA (UVB) group; 31.3 ± 0.9 μm in the eyes of GGA-treated but non-irradiated mice (GGA); and 33.7 ± 0.6 μm in the eyes of naive mice. Corneal epithelia were significantly thicker in eyes treated with GGA compared with vehicle-given eyes after irradiation (*p* < 0.01, Figure 1E).

**Figure 2:**
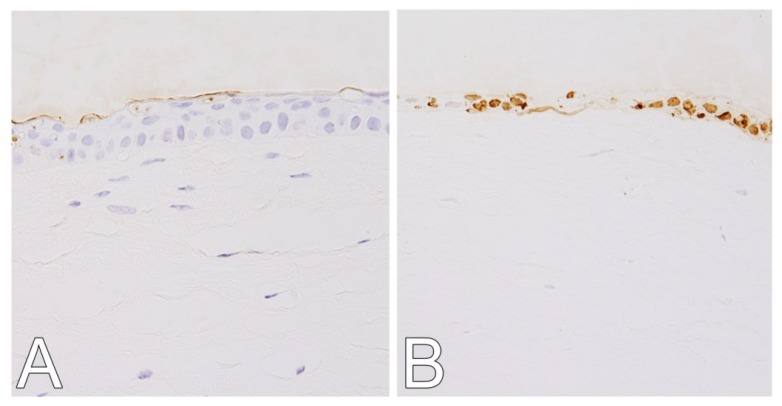

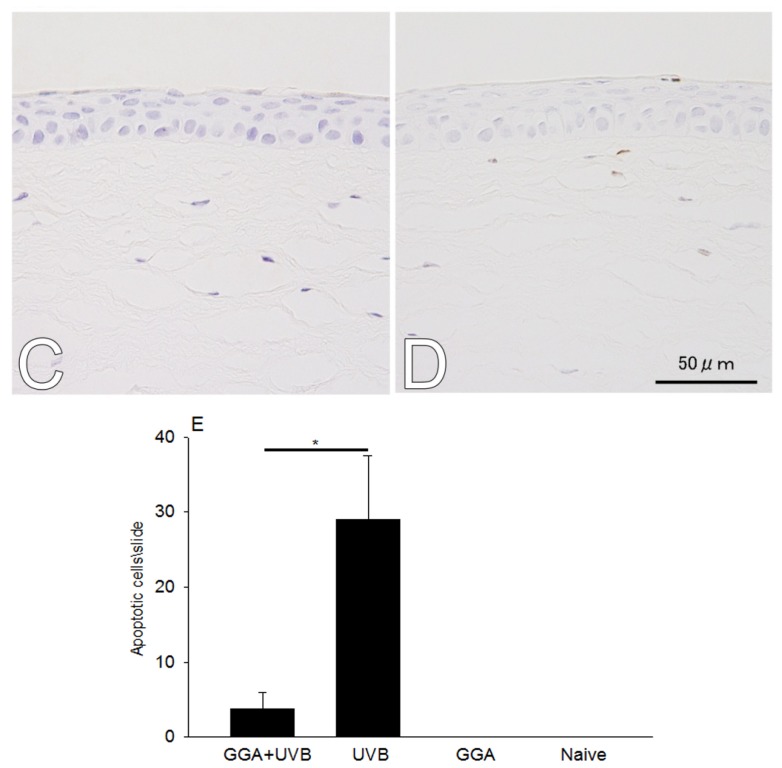
Number of terminal deoxynucleotidyl transferase dUTP nick end labeling (TUNEL) positive corneal cells after of ultraviolet (UV) B exposure. (**A**) Irradiated eyes of mice treated with geranylgeranylacetone (GGA). (**B**) Irradiated eyes of mice without GGA administration. (**C**) Unirradiated eyes of mice administered with GGA. (**D**) Naive corneas. (**E**) Mean number of terminal deoxynucleotidyl transferase dUTP nick end labeling-positive nuclei. Data are shown as mean ± SEM (*n* = 10), *p < 0.05 (Mann-Whitney U-Test).

Cell-death response was decreased in corneas of the GGA + UVB group (Figure 2A) compared with the vehicle-given mice UVB group (Figure 2B) where multiple terminal deoxynucleotidyl transferase dUTP nick end labeling (TUNEL)-positive cells were detected. There were no apoptotic cells detected in corneas not exposed to UVB (Figure 2C,D). The mean numbers of TUNEL-positive cells in mice with irradiated corneas were only (3.75 ± 2.2)/slide in the eyes of mice treated with GGA (GGA + UVB), by contrast 29.0 ± 8.5 in the eyes of vehicle-given mice (UVB). After irradiation, there were significantly fewer apoptotic cells in the corneas of mice given GGA than those receiving vehicle alone (*p* < 0.01, Figure 2E).

### 2.2. Effect of GGA on HSP70 Expression

**Figure 3:**
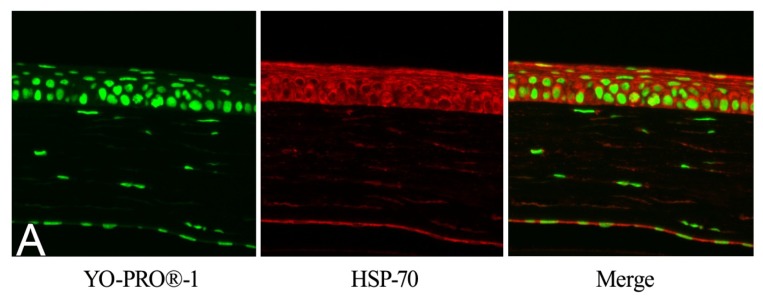

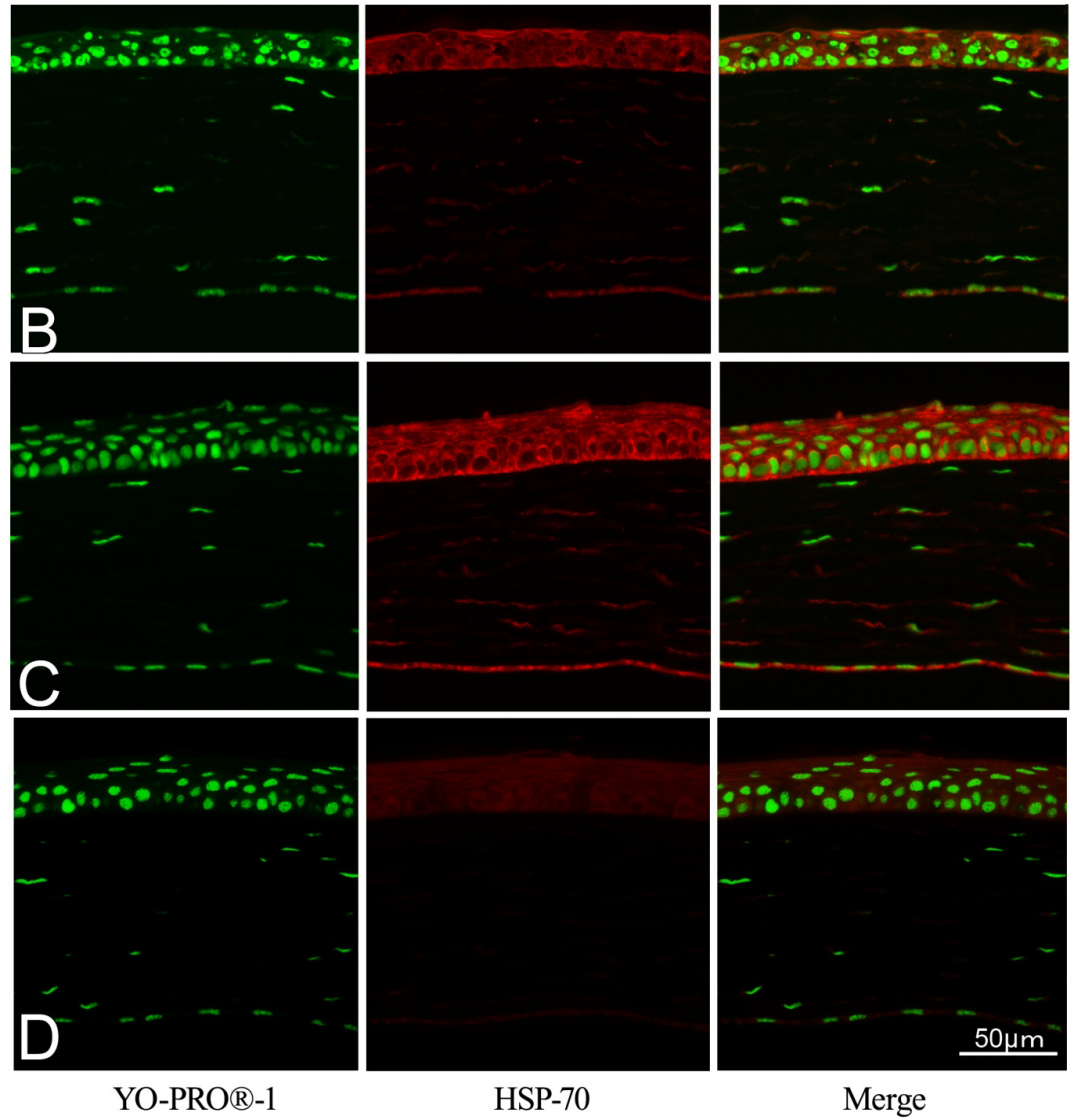
Immunohistochemical evaluation of heat shock protein 70 expression after treatment with geranylgeranylacetone and ultraviolet B exposure. (**A**) Irradiated eyes of mice treated with geranylgeranylacetone (GGA). (**B**) Irradiated eyes of mice without GGA. (**C**) Unirradiated eyes of mice administered with GGA. (**D**) Naive corneas.

HSP70 were detected in the corneas of GGA-treated mice after UVB irradiation (Figure 3A), GGA-untreated irradiated mice (Figure 3B), and GGA-given unirradiated mice (Figures 3C). HSP70 was induced in cytoplasm and nuclei in these three groups. However, low a HSP70 signal was observed in naive controls (Figure 3D).

### 2.3. Quantification of HSP70 Levels

**Figure 4:**
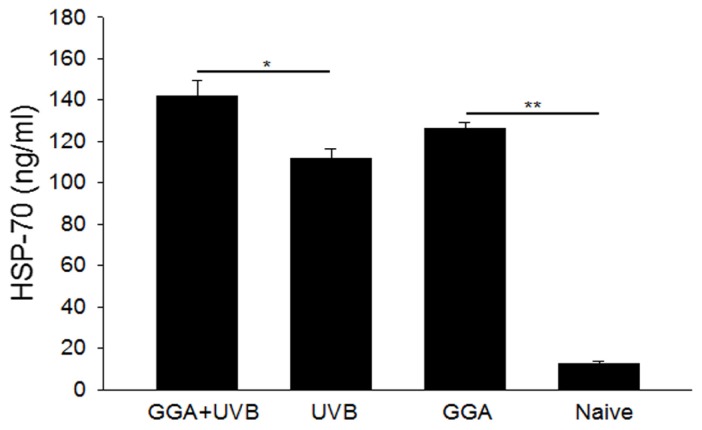
Enzyme-linked immunosorbent assay (ELISA) evaluation of HSP70 expression after treatment with geranylgeranylacetone and ultraviolet B exposure. Data are shown as mean ± SEM (*n* = 4), **p < 0.01; *p < 0.05 (Mann-Whitney U-Test).

Next, corneal HSP70 levels were quantified with enzyme-linked immunosorbent assay (ELISA). GGA administration significantly increased HSP70 concentrations in corneal epithelia of GGA + UVB mice with 142.13 ± 7.3 ng/mL compared with those of the UVB group not treated with GGA with 111.77 ± 4.8 ng/mL (*p* < 0.05). Even when mice were not irradiated, HSP70 expression was also significantly increased in the GGA-treated group (126.44 ± 2.8 ng/mL) compared with those of naive subjects (12.4 ± 1.5 ng/mL, *p* < 0.01). Considerable production of HSP70 in the corneal tissue was induced in the GGA-treated (GGA+UVB and GGA) groups (Figure 4).

### 2.4. Detection of ROS

**Figure 5:**
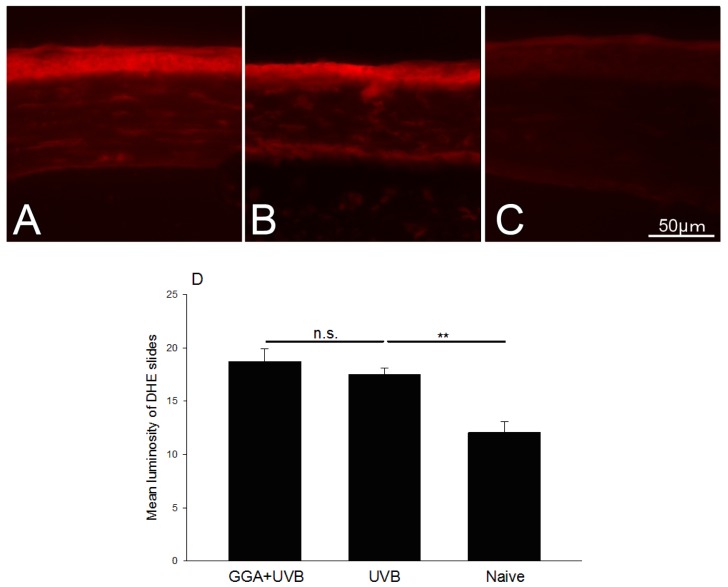
Reactive oxygen species (ROS) signal expression after UVB exposure. (**A**) Eyes of mice treated with 500 mg/kg GGA and irradiated at a dose of 400 mJ/cm^2^. (**B**) Eyes not treated with GGA and irradiated at a dose of 400 mJ/cm^2^. (**C**) Naive corneas. (**D**) Mean gray values of the corneal epithelium of DHE stained slides. **p < 0.01; n.s. *p* > 0.05 (Mann-Whitney U-Test).

Reactove oxygen species (ROS) expression was examined in the corneal epithelium of irradiated mice treated with GGA (Figure 5A), exposed to UVB only (Figure 5B), and naive mice (Figure 5C). The mean gray values of the corneal epithelium on dihydroethidium (DHE) stained slides were evaluated by the Image J software and are summarized in Figure 5D. Mean gray values were 18.73 ± 2.0, 17.48 ± 1.0, and 12.04 ± 1.7, in mice irradiated with GGA, irradiated without GGA, and naive mice, respectively. ROS production was significantly increased after irradiation regardless of GGA administration. GGA had little effect on ROS production.

### 2.5. NF-κB Immunohistochemistry Staining

**Figure 6:**
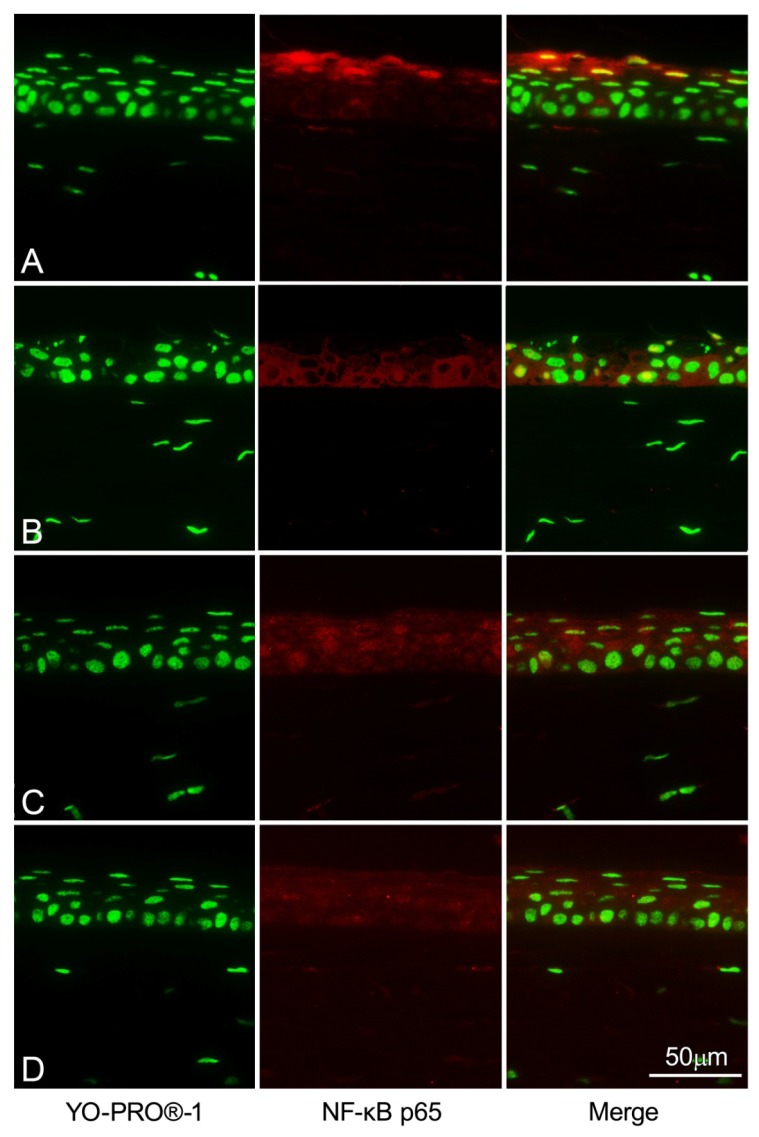
Immunohistochemical evaluation of NF-κB expression after treatment with GGA. (**A**) Eyes of mice treated with GGA and irradiated. (**B**) Eyes with no GGA but irradiated. (**C**) Eyes of mice treated with GGA but not irradiated. (**D**) Naive corneas.

To examine whether GGA administration affects NF-κB in corneal epithelium, we immunohistochemically analyzed NF-κBp65 translocation with a confocal microscope. NF-κB positive nuclei (yellow) were rarely seen in corneal epithelial cells in UV-irradiated mice treated with GGA 500 mg/kg except for some keratinized cells (Figure 6A). However multiple NF-κB positive nuclei were detected in corneal epithelial cells in UV-irradiated mice untreated with GGA (Figure 6B). Corneal tissues without UVB irradiation showed a weak cytoplasmic response to anti-NF-κB antibodies with no nuclear colocalization observed. (Figure 6C,D).

### 2.6. Effect of GGA on Akt Phosphorylation

**Figure 7:**
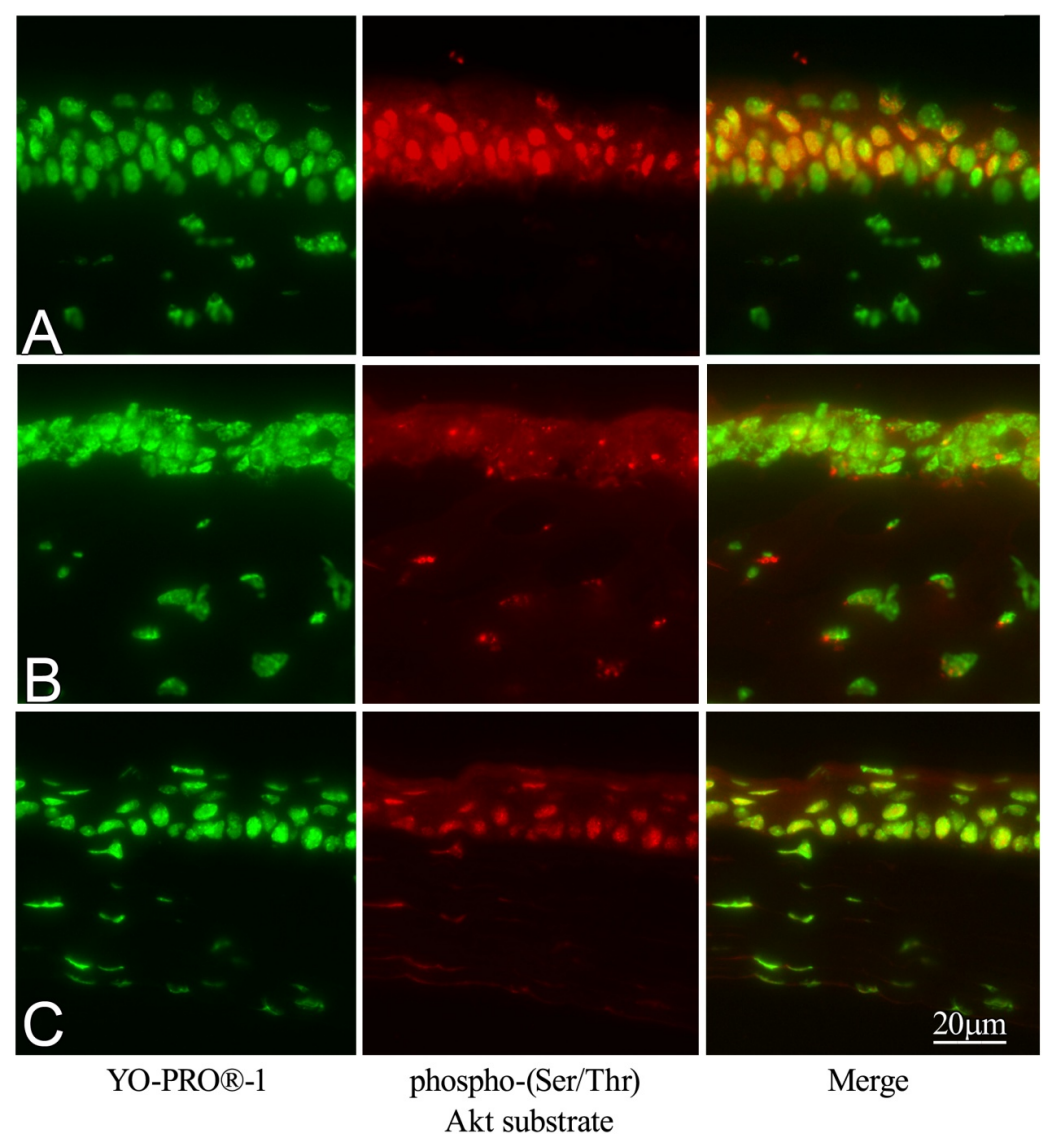
Immunohistochemical evaluation of phospho-(serine/threonine) Akt substrate expression after treatment with geranylgeranylacetone and ultraviolet B exposure. (**A**) Irradiated eyes of mice treated with GGA. (**B**) Irradiated eyes without GGA. (**C**) Naive corneas.

Markedly increased Phospho-(Ser/Ther) Akt substrate was observed in irradiated corneas when mice were treated with GGA to induce HSP70 (Figure 7A). However, when untreated with GGA, little phosphorylated Akt expression was detected in corneal epithelium (Figure 7B). A certain phosphorylated Akt immunolabeling of corneal epithelium was also observed in naive mice (Figure 7C).

## 3. Discussion

The corneal epithelium protects eye structures against UV damage by absorbing a substantial part of the UV energy applied to the ocular surface. In the present study, it was shown that UVB-induced corneal damage was ameliorated by oral administration of GGA, and HSP70 was markedly elevated in corneas. The possible molecular mechanisms of GGA following HSP70 induction are the following: (1) Inhibition of inflammatory pathways, (2) suppression of oxidative stress, and/or (3) activation of cell survival signals.

As other investigators and we have reported, UVB induces excessive ROS production in the cornea and affects several cellular pathways, including the activation of nuclear factor-κB (NF-κB) and other pro-inflammatory mediators as well as direct DNA and protein damage [1,2,15]. However, GGA administration ameliorated UVB-induced corneal damage without affecting ROS production but increased the expression of HSP70 in the corneal tissue in the present study. Several recent reports have indicated that increased HSP70 expression reduces NF-κB activation *in vivo* [16] and *in vitro* [17,18] without ROS suppression, and one of the proposed mechanism of the phenomenon is the interaction with TNF receptor associated factor (TRAF6) and the inhibition of its ubiquitination [17]. Other previous studies have shown that corneal NF-κB activation is necessary for the retention of transparency in the cornea of UVB-exposed mice [19], and a proinflammatory cytokine, macrophage migration inhibitory factor (MIF), up-regulated NF-κB [20]. In fact, we reported more corneal damage in MIF over-expressing mice than in the wild type, but less corneal damage in MIF knockout mice than in the wild type [21]. Although it has long been speculated that proinflammatory cytokines may play an important role in the wound repair of ocular tissue, this mechanism has never been fully understood. Since our present immunohistochemical findings showed a decrease of NF-κB nuclear co-localization in UVB irradiated corneas after GGA treatment, in a way, the milder corneal damage when treated with GGA should owe its HSP70 induction to the suppression of inflammatory signals, not to deoxidation.

The third possible effective pathway of GGA administration may be the activation of cell survival signals. It may lead to cell survival under stress condition by GGA administration by increased Akt phosphorylation by HSP70. In this study, a marked phospho-(Ser/Ther) Akt substrate expression was observed in irradiated corneal epithelium when mice were pre-treated with GGA. The results were consistent with previous studies reporting the association of GGA/HSP70 and Akt/caspase signals in retinas of the eye [13]. Thus, induced HSP70 by GGA administration suppresses cell death signals by reducing NF-κB activation, and possibly activates cell survival pathway Akt phosphorylation. These newly identified mechanisms may contribute to our understanding of photo-induced ocular damage.

A small amount (0.2%) of vitamin E was contained as additive in the solution in this study. Previous studies have reported that vitamin E itself has a certain level of anti-inflammatory effect in eyes [22]. Though 150 mg/kg of vitamin E was reported to be effective in an ocular inflammation model, only 1 mg/kg of vitamin E was applied to mice along with the GGA solution in our current study. The vitamin E contained in the GGA solution was 150 times lower than the reported effective dose. Moreover the protective features of vitamin E are mostly attributed to its antioxidant activity [23]. Our ROS (DHE)-staining section slides never showed significant reduction of ROS production in the vehicle group. So it is expected that the ameliorative effect of the cornea against UV is mostly attributed to GGA, not to vitamin E additives. The present results in mice allow us to speculate about a similar effect of HSP70 in humans.

For future clinical application, post-UV treatment of GGA may be preferable. However, this model took around 24 h to metabolize and to elevate HSP70 expression *in vivo*. Also, it showed severe corneal damage/epithelial cell death immediately after UV exposure, and eyes should be collected 24 h after irradiation. Further studies are required, using another design to examine the effectiveness of post-UV treatment with GGA.

## 4. Experimental Section

### 4.1. Animals and Reagents

Six- to eight-week-old C57BL/6 male mice were obtained from CLEA Japan (Tokyo, Japan). Mice were maintained under specific pathogen-free (SPF) conditions. All procedures involving animals were performed in accordance with the Association for Research in Vision and Ophthalmology (ARVO) resolution on the use of animals in research. GGA and gum arabic were provided by Eisai Co., Ltd (Tokyo, Japan). GGA was emulsified with 0.5% gum arabic and distilled water containing 0.2% vitamin E. Unless stated otherwise, all experiments were performed in triplicate, involving at least five animals (10 eyes) per group.

### 4.2. UV Irradiation and Sample Collection

Four groups of C57BL/6 mice were established in the experiment; the GGA + UVB group was orally administered GGA (500 mg/kg body weight) by using feeding needles, while the control group (UVB) received the vehicle alone. UV-photokeratitis was induced as reported recently [15]. Twenty-four hours after GGA pre-treatment, anesthetized mice were irradiated with UVB at a dose of 400 mJ/cm^2^ from a FS-20 (Panasonic, Osaka, Japan) fluorescent lamp. To examine HSP70 expression, corneas were irradiated with 200 mJ/cm^2^. These bulbs have a broad emission spectrum (250–400 nm) with a high output, primarily in the UVB spectrum (290–320 nm). Two additional groups without UVB irradiation were used, the “GGA” group was orally administered GGA (500 mg/kg body weight) to evaluate the effects of GGA on the corneal surface without UVB damage, and “naive” mice were used as controls. Euthanasia was performed by intraperitoneal injection of sodium pentobarbital (Sigma, Tokyo, Japan, 100 mg/kg). Eyeballs were collected 24 h after irradiation and stained with hematoxylin-eosin (H&E), terminal deoxynucleotidyl transferase dUTP nick end labeling (TUNEL), anti-HSP70 antibody [24] and anti-phospho-(Ser/Ther) Akt substrate antibodies (Cell Signaling Technology, Tokyo, Japan).

### 4.3. H&E and TUNEL Staining

The eyes were dissected from the mice 24 h after UVB exposure and fixed with 4% paraformaldehyde and embedded in paraffin, tissue sections were prepared and stained with H&E for morphological analysis. At least eight sections were used to evaluate the epithelial thickness by performing measurements of 10 randomly selected areas of epithelium of the central cornea and the results were averaged. Other sections were stained by terminal deoxynucleotidyl transferase dUTP nick end labeling (TUNEL) assay to detect cell death, with a cell death detection kit (Roche Diagnostics Japan, Tokyo, Japan) containing all necessary reagents for staining. Slide imaging, cell counting, and thickness evaluations were performed with a BZ-9000 fluorescence microscope (Keyence, Osaka, Japan).

### 4.4. Immunohistochemical Staining for HSP70 and Akt Substrate in Corneal Tissue

GGA induced HSP70 expression in the retinas of mice [7], and we checked HSP70 induction in the corneas. The eyes were dissected from mice 24 h after UVB exposure and fixed with 4% paraformaldehyde and then paraffin-embedded. To evaluate the HSP70 expression in the corneal epithelium, slides were rehydrated and blocked with bovine albumin 5% solution 30 min and then a 1:500 dilution of anti-HSP70 antibody was applied for 12 h, washed with phosphate buffered saline (PBS) (NaCl 8 g, (Na_2_HPO_4_)_12_H_2_O 2.9 g, KCl 0.2 g, KH_2_PO_4_ 0.2 g, H_2_O 1 L; pH 7.4), and then 1:1000 dilution of secondary goat anti-rabbit 546 nm antibody dye conjugate (Invitrogen, Carlsbad, CA, USA) was applied, as described previously [7]. Anti-HSP70 antibody was developed as follows: Recombinant human HSP70 (amino acids 1–641) fused to glutathione-*S*-transferase was immunized into rabbits in a TiterMax (CrtRx Co., Georgia) water-in-oil emulsion. Antiserum specific for HSP70 was generated and the antibody showed great specificity [24]. Cell nuclei were stained with 1:1000 dilution of YO-PRO^®^ (Invitrogen).

Some histological sections were prepared as described above and then a 1:100 dilution of phospho-(Ser/Thr) Akt substrate antibody (Cell Signaling) was applied for 24 h. Sections were then rinsed again with PBS and secondary 546 nm goat anti-rabbit antibody dye conjugate (Invitrogen). Cell nuclei were stained with 1:1000 dilution of YO-PRO^®^ (Invitrogen).

### 4.5. Quantifying HSP70 with Enzyme-Linked Immunosorbent Assay (ELISA)

Eyes were enucleated 24 h after UVB irradiation and 48 h after GGA treatment. Corneal tissues were carefully dissected from the eyes under the microscope and emulsified with 500 μL of radioimmunoprecipitation assay (RIPA) buffer with a protease inhibitor cocktail (Roche, Penzberg, Germany), then centrifuged at 20,000*g* for 20 min at 4 °C. Total protein concentration was adjusted using a bicinchoninic acid (BCA) protein assay kit (Pierce Biotechnology, Rockford, IL, USA). The HSP70 concentration was evaluated with a HSP70 high-sensitivity ELISA kit (Enzo Life Sciences, New York, NY, USA). All experiments were performed in triplicate with four or more wells for each group in each experiment.

### 4.6. Detection of Reactive Oxygen Species (ROS)

The eyes were dissected from mice 24 h after UVB exposure and fresh frozen at optimal cutting temperature (OCT) compound with liquid nitrogen. Dihydroethidium (DHE; Sigma-Aldrich, St. Louis, MO, USA), an oxidative fluorescent dye, was used for the immunohistochemical detection of cytosolic superoxide anion (O_2_-) to evaluate ROS production in corneal epithelium tissue, as reported recently [15,25]. Confocal microscopic images of ROS production were examined in mouse corneal tissue. ROS production was determined by conversion of DHE to ethidium bromide (EtBr). All images were made in parallel at identical settings.

### 4.7. NF-κB Immunohistochemistry Staining

The eyes were dissected from mice 24 h after UVB exposure and fixed with 4% paraformaldehyde and then paraffin-embedded. To evaluate the NF-κB positive cells in the corneal epithelium, slides were rehydrated and a 1:50 dilution of NF-κB p65 antibody (Santa Cruz Biotechnology, Santa Cruz, CA, USA) was applied for 12 h, then washed with PBS, and a secondary goat anti-rabbit antibody dye conjugate (Invitrogen) with a 1:1000 dilution was applied. NF-κB is widely present in intercellular tissues. To detect expression of NF-κB in cell nuclei, slides were stained with 1:1000 dilution of YO-PRO®-1 (Invitrogen), and colocalization of NF-κB (yellow) in nuclei was observed in merged images.

### 4.8. Statistical Analysis

All values were expressed as the mean ± standard error of mean (SEM) from the respective groups of experimental or control data. Statistical significance was evaluated by using non-parametric Mann-Whitney U Test. *p* values less than 0.05 were considered significant.

## 5. Conclusions

GGA administration induces HSP70 in cornea under stress condition and limits the corneal cell damage by UVB exposure to reduce NF-κB activation and possibly to suppress phosphorylation of Akt, a cell survival signal. Considering that GGA is now well established in the treatment of stomach ulcers, it may also be a promising supplemental treatment for protecting ocular surfaces from UV-photo damage.
